# Molecular detection and characterization of *Theileria* spp. infecting cattle in Sennar State, Sudan

**DOI:** 10.1007/s00436-018-5775-0

**Published:** 2018-03-07

**Authors:** Sofia B. Mohamed, Atif Alagib, Tahani B. AbdElkareim, Mohamed M. Hassan, Wendell C. Johnson, Hala E. Hussein, Naomi S. Taus, Massaro W. Ueti

**Affiliations:** 1 National University Research Institute, Khartoum, Sudan; 2 Tropical Medicine Research Institute, Khartoum, Sudan; 3 Medical Laboratory Sciences, Al Zaiem Al Azhari University, Khartoum, Sudan; 4 USDA-ARS-Animal Disease Research Unit, Pullman, WA USA; 5 Department of Entomology, Faculty of Science, Cairo University, Giza, Egypt; 6 Department of Veterinary Microbiology and Pathology, Washington State University, Pullman, WA 99164-7040 USA

**Keywords:** *Theileria* spp., 18S rRNA gene, PCR, Coinfection, Cattle, Sudan

## Abstract

Tropical theileriosis is a serious animal disease transmitted by tick vectors. The agents of theileriosis are obligate intracellular parasites that cause mild to severe disease in the mammalian host. Tropical theileriosis has been recognized as a burden to the development of the dairy industry in Sudan and causes major economic losses. However, knowledge about the distribution of *Theileria* spp*.* in Sudan and the extent of sequence variation within the 18S rRNA gene is currently unknown. The aim of this study was to determine the diversity of *Theileria* spp. using 18S rRNA-based PCR to detect parasites in cattle followed by cloning and sequencing. We observed an overall prevalence rate of 63% hemoparasite infection in cattle from Sennar state. A subset of samples was used for cloning and sequencing of the 18S rRNA gene. Nineteen of 44 animals were co-infected with more than one species of *Theilera*. Phylogenetic analysis revealed three *Theileria* spp. that were predominant in cattle including pathogenic *T. annulata* and apathogenic *T. velifera* and *T. mutans*. The present study provides information regarding the prevalence of theileriosis in Sudan and will help to design strategies to control it. Additionally, more study is needed to determine tick vector competence and degree of coinfection with multiple *Theileria* spp. in Sudan. This represents the first molecular phylogeny report to identify *Theileria* spp. in cattle from Sudan.

## Introduction

Tropical theileriosis is a serious and widespread disease transmitted by ticks. Theileriosis leads to major economic losses for the livestock industry (Brown 1990). In Khartoum State, Sudan, more than 80% of farms experienced clinical theileriosis resulting in significant economic losses (El Hussein et al. 2012). This disease is caused by the apicomplexan *Theileria annulata* and found primarily in tropical and subtropical regions of the world (Mhadhbi et al. 2010). Approximately 250 million animals are living in constant risk of acquiring theileriosis. *T. annulata* parasites are transmitted by *Hyalomma anatolicum* (El Hussein et al. 2012). This parasite has been detected in field collected *H. anatolicum* ticks from central and northern Sudan implicating it as an important vector. Strategies to control tropical theileriosis are controlling tick infestation by acaricides, immunization using live vaccines, and treatment of infected cattle (Akat et al. 2014). Early studies in Sudan based on Giemsa-stained blood and lymph node biopsy smears detected widespread and high prevalence of *Theileria* spp. infection (El Hussein et al. 2012). Previous studies using indirect fluorescent antibody (IFA) tests detected prevalence ranging from 18 to 90% (El Hussein et al. 2012). In another study using enzyme-linked immunosorbent assay (ELISA) seroprevalence in Sudan ranged from 6 to 86% (Guma et al. 2015). Studies using polymerase chain reaction (PCR) and reverse line blot (RLB) revealed *T. annulata* prevalence rates in dairy cattle of 48 and 65%, respectively (Ali et al. 2006).

Advances in sequence data analysis have allowed researchers to identify and characterize hemoparasites, in particular members of the *Theileria* genus. Ribosomal RNA is the most abundant constituent of nucleic acids in any non-viral organism with the eukaryotic RNA transcription unit consisting of the 28S, 18S, and 5.8S rRNAs (Waters and McCuthan 1990). The conserved function and structure of 18S rRNA allow sequences to be aligned, even among divergent species. However, the molecule also possesses phylogenetically informative variable regions that are useful for determining relationships among species (Hillis and Dixon 1991). Cloning and sequencing of the 18S rRNA gene have been used to study the diversity of *Theileria* spp. in cattle in parts of Asia, Europe, and Africa (Brígido et al. 2004; Criado-Fornelio et al. 2003, 2009; Gebrekidan et al. 2014; Liu et al. 2010; Mans et al. 2011). However, the taxonomy and characterization of the18S rRNA gene in cattle *Theileria* spp. have not yet been defined in Sudan. This study was conducted using molecular genetic techniques involving PCR, cloning, and sequencing of an 18S rRNA gene fragment to detect and characterize species of *Theileria* infecting cattle in Central Sudan. The present study provides insights into the epidemiology of tropical theileriosis which will help to design control strategies in Sudan.

## Materials and methods

**Fig. 1:**
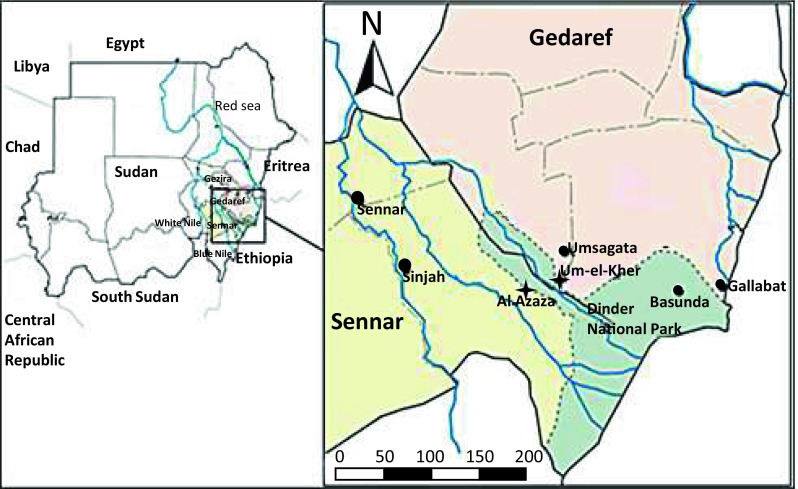
Study sites were located in Sennar State, shown in yellow

This study was conducted in Sennar state located in Central Sudan between latitudes 12.5–14.7 ^°^N and longitudes 32.9–35.4 ^°^S, bordered by Gezira State to the north, White Nile State to the west, Gedaref State to the east, and Blue Nile and Upper Nile States to the south with an area of 40.680 km^2^ (Fig. 1). Whole blood was collected from 180 cattle from 11 sites within Sennar State (Table 1). The samples were collected on filter paper (GE Healthcare, Germany) and DNA isolated using QIAamp DNA Mini Kit according to the manufacturer’s instructions (Qiagen, Germany).

**Table 1:** Detection of hemoparasites in cattle in Sennar State, Sudan

Location	PCR positive	% detection
Tayba Elhafer	9/14	64.3%
Ellukha	20/23	87%
Suliman farmer	8/13	61.5%
Banta	15/20	75%
Elnifdya	17/21	81%
East Sennar	13/22	59%
West Sennar factory	6/12	50%
East Sugar factory	12/22	54.6%
Tabat Alojba	4/10	40%
Alamaria	5/8	63%
Alamaria 64	4/15	27%
Total	113/180	63%

Genomic DNA isolated from whole blood was used as the template in a nested PCR. Analysis of 18S rRNA gene among tick-borne protozoan parasites showed that these primer sets flanking a hypervariable region can detect *Theileria* spp. and *Babesia* spp. Outer primers (Nbab-1 forward, 5′-AAG CCA TGC ATG TCT AAG TAT AAG CTTTT-3′ and Nbab-1 reverse, 5’-CTT CTC CTT CCT TTA AGT GAT AAG GTT CAC-3′) amplify a fragment of 1600 bp (Oosthuizen et al. 2008). PCR was carried out under the following conditions: 95 °C for 5 min; 35 cycles of 95 °C for 30 s, 50 °C for 30 s, and 72 °C for 1 min; final extension at 72 °C for 5 min. The reaction was conducted in 32 μl containing 2 μl of extracted genomic DNA, 2.0 mM MgCl_2_, 200 μM dATP, dCTP, dGTP, dTTP, 1.0 μM of each primer set, and 1.3 U of FastStart Taq (Roche, USA). The 18S rRNA inner primers (forward, 5′-AAT CCT GAC ACA GGG AGG TAG TGA C-3′ and reverse, 5’-CTA AGA ATT TCA CCT CTG ACA GT-3′) amplify a fragment of 390 bp (Ueti et al. 2015). Nested PCR was carried out under the following conditions: 95 °C for 5 min; 35 cycles of 95 °C for 30 s, 65 °C for 30 s, and 72 °C for 30 s; final extension at 72 °C for 5 min. The reaction was conducted in 30 μl containing 0.1 μl from the first reaction, 2.0 mM MgCl_2_, 200 μM dATP, dCTP, dGTP, dTTP; 1.0 μM of each primer set, and 1.25 U of FastStart Taq (Roche). Amplification was performed in an automated thermocycler (Bio-Rad, USA).

To determine presence of *Theileria* in Sennar, a subset of samples from each location was used for cloning and sequencing a portion of the 18S rRNA hypervariable region. A previous study (Mans et al. 2016) demonstrated that the number of *Theileria* spp. detected in the herd population by next-generation sequencing techniques was similar to that found by conventional PCR (Mans et al. 2011). Therefore, in this study, PCR products were cloned and sequenced to determine *Theileria* diversity from cattle in Sennar. Nested PCR products were cloned into pCR® II-TOPO® (ThermoFisher Scientific, USA), transformed into competent *Escherichia coli* TOP10 cells, and cultured in antibiotic selection media based on manufacturer’s guidelines (ThermoFisher Scientific). Five colonies per amplification from each sample were randomly selected, and purified plasmids were sequenced using 150 ng plasmid DNA and 5 pmol M13 forward and M13 reverse primers as well as the18S rRNA inner gene specific primers for the sequencing procedure by Eurofins MWG Operon (Huntsville, USA). A total of 220 clones were sequenced, edited, and assembled to a final length of ~ 400 bp.

All reference sequences of the 18S rRNA genes of *Theileria* used in this study were obtained from GenBank (https://www.ncbi.nlm.nih.gov/genbank/) using the blastn algorithm (https://blast.ncbi.nlm.nih.gov). DNA sequence alignments were performed by MUSCLE v. 3.8.31 on the European Bioinformatics Institute (EBI) homepage (http://www.ebi.ac.uk/Tools/msa/muscle). The evolutionary history was inferred using the neighbor-joining method. The tree is drawn to scale, with branch lengths in the same units as those of the evolutionary distances used to infer the phylogenetic tree. The evolutionary distances were computed using the maximum composite likelihood method and are in the units of the number of base substitutions per site. All positions containing gaps and missing data were eliminated. Tree construction and evaluation were performed using PhyML with the approximate likelihood-ratio test for branch support located at the phylogeny.fr webserver (http://www.phylogeny.fr/) (Dereeper et al. 2008; Dereeper et al. 2010).

## Results

**Fig. 2:**
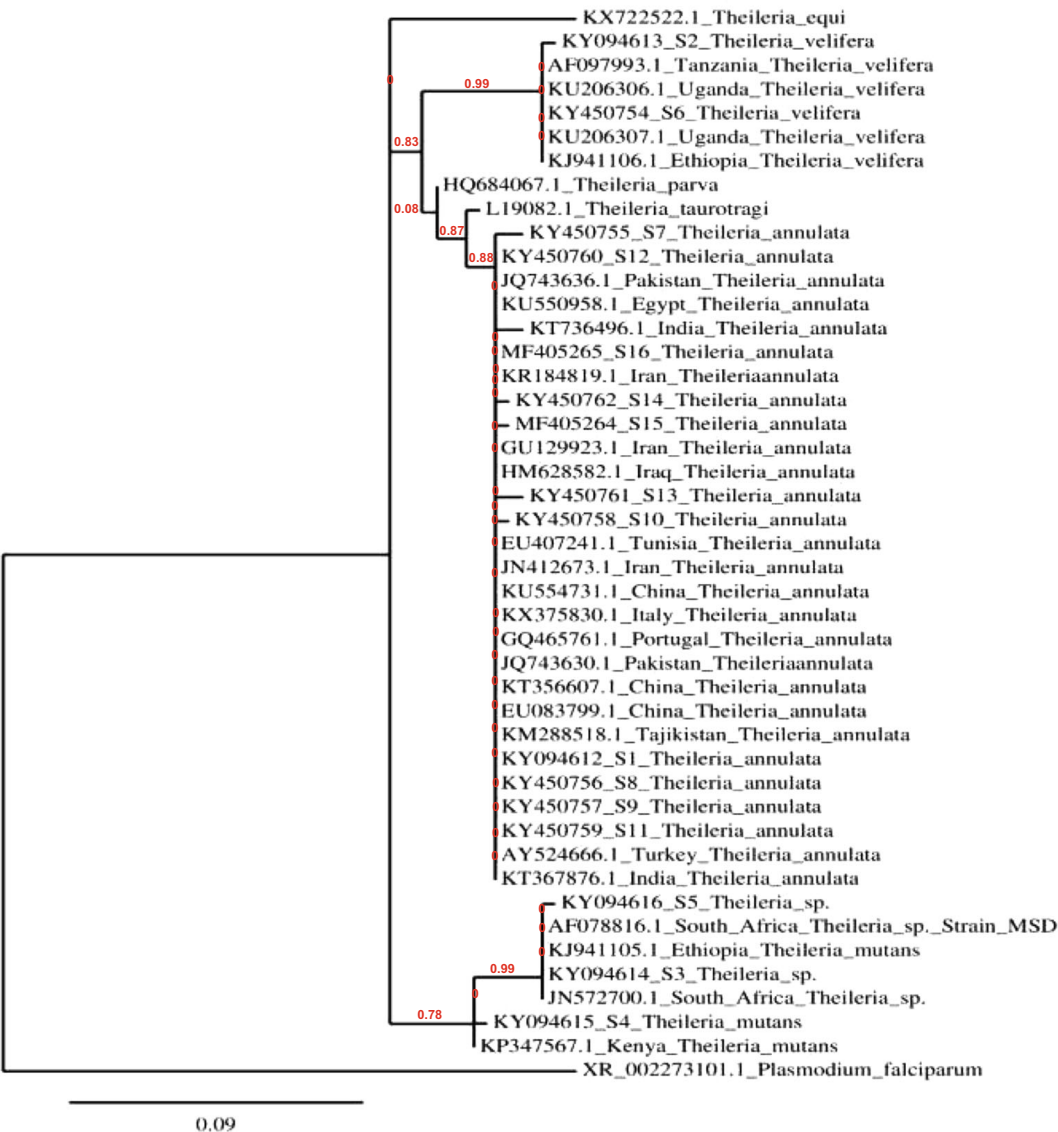
Neighbor-joining analysis of the 18S rRNA sequences showing the phylogenetic relationship of the *Theileria* spp. Branch lengths are proportional to the estimated genetic distance between species. Sequences are shown as GenBank accession number location *Theileria* sp. S1–16 are the Sudan sequences from this study

\Hemoprotozoan parasite-specific PCR revealed that 113 out of 180 (63%) cattle were infected. The highest prevalence of infection was found in Elnifdya town (81%) and the lowest prevalence in Alamaria 64 (27%). The prevalence of infection in different Sennar areas is shown in Table 1. Cloning and sequencing of the 18S rRNA PCR products from a subset of 44 randomly selected cattle revealed 16 *Theileria* sequences, S1-S16, which were deposited in GenBank (Table 2 and Fig. 2). Infection with only *T. annulata* was detected in 50% (22/44) of the cattle. Coinfection of cattle with *T. annulata* and either *T. mutans* (14/44) or *T. velifera* (8/44) was also revealed. Phylogenetic analysis demonstrated that *Theileria* spp. present in the Sennar cattle population are closely related to *Theileria* from several parts of the world (Fig. 2 and Table 2).

**Table 2:** Identification of *Theileria* species infecting cattle in Sudan

Sequences (accession #)	% identity	Organism	Location (accession number)
S1 (KY094612)	99	*T. annulata*	India (KT367876.1); Egypt (KU550958.1)
S2 (KY094613)	99	*T. velifera*	Uganda (KU206307.1/KU206306.1); Ethiopia (KJ941106.1); Tanzania (AF097993.1)
S3 (KY094614)	99	*T. annulata*	Iran (JN412673.1)
	99	*Theileria* sp*. MNM-2014a*	Turkey (KF916506.1)
S4 (KY094615)	100	*T. mutans*	Ethiopia (KJ941105.1); Kenya KP347567.1)
S5 (KY094616)	100	*Theileria mutans MSD*	South Africa (JN572700.1)
	99	*Theileria mutans MSD*	South Africa (AF078816.1/KU206308.1)
S6 (KY450754)	99.9	*T. velifera*	Uganda (KU206307.1/KU206306.1); Ethiopia (KJ941106.1); Tanzania (AF 097993.1)
S7 (KY450755)	99	*T. annulata*	Iraq (HM628582.1)
S8 (KY450756)	99	*T. annulata*	Turkey (AY524666.1); Tajikistan (KM288518.1)
S9 (KY450757)	99	*T. annulata*	India (KT736496.1); Tunisia (EU407241.1); China (EU083799.1)
S10 (KY450758)	99	*T. annulata*	China (KU554731.1, KT356607.1); Egypt (KU550958.1)
S11 (KY450759)	99	*T. annulata*	Iran (GU129923.1)
S12 (KY450760)	99	*T. annulata*	Turkey (AY524666.1); Pakistan (JQ743630.1)
S13 (KY450761)	99	*T. annulata*	Iran (KR184819.1); Portugal (GQ465761.1)
S14 (KY450762)	99	*T. annulata*	India (KT367876.1)
	99	*Theileria sp. MNM-2014a*	Turkey (KF916506.1)
S15 (MF405264)	99	*T. annulata*	Italy (KX375830.1)
S16 (MF405265)	99	*T. annulata*	Pakistan (JQ743636.1)

## Discussion

In Sudan, there are few reports showing the incidence or prevalence of *Theileria* infection using molecular-based assays particularly with regard to *Theileria* spp. other than *T. annulata* (El Hussein et al. 2012). Additionally, coinfections with multiple *Theileria* spp. in cattle in Sudan have not been reported. Unlike the RLB, cloning and sequencing the 18S rRNA gene allow direct determination of the diversity among *Theileria* spp. present in the cattle population as well as detection of coinfections (Mans et al. 2011). A previous study (Mans et al. 2016) demonstrated that the number of *Theileria* spp. detected in the herd population using next generation sequencing was similar to conventional PCR results (Mans et al. 2011). In this study, we found a high prevalence of hemoprotozoan parasite infections in cattle in Sennar. However, PCR amplification did not discriminate infection between *Theileria* spp. and *Babesia* spp. Sequence analysis of the 18S rRNA gene from a subset of samples revealed infection with pathogenic *T. annulata* as well as apathogenic *T. mutans*, *T. mutans* MSD, and *T. velifera*. The lack of clinical disease suggests that infected animals were in the chronic phase of infection. These infected animals are reservoirs for tick transmission.

Transmission of hemoprotozoan parasites is optimally maintained when competent tick vectors and infected animals are found together. In Sudan, *H. anatolicum* is the vector for *T. annulata* (El Hussein et al. 2012). Detection of apathogenic *T. mutans* and *T. velifera* in Sudan herds indicates the presence of *Amblyomma* spp., particularly *A. variegatum*, a known vector for both of these *Theileria* spp. (Purnell et al. 1975).

The impact of apathogenic-mixed infection on the severity of tropical theileriosis caused by *T. annulata* warrants further investigation. A previous study demonstrated that mixed infection with apathogenic *Theileria* reduced mortality associated with *T. parva* infection (Woolhouse et al. 2015).

The 18S rRNA primer sets used in this study were designed to detect protozoan parasites including *Theileria* and *Babesia* parasites. One interesting finding in this study is all sequences matched with *Theileria* spp. Sequence analysis for 220 clones showed no evidence of *Babesia* infection. A possible explanation for the failure to detect *Babesia* spp. is the level of parasites in the peripheral blood was below the threshold of PCR detection (Suarez et al. 2012).

In conclusion, the high prevalence of *Theileria annulata* infection in the cattle population supports that Sudan is endemic for tropical theileriosis, a devastating tick-borne disease of the cattle industry. Understanding the epidemiological distribution of pathogenic and apathogenic parasites in herds will allow development of strategies to control tropical theileriosis in Sudan.
